# Electronic Health Record Portal Messages and Interactive Voice Response Calls to Improve Rates of Early Season Influenza Vaccination: Randomized Controlled Trial

**DOI:** 10.2196/16373

**Published:** 2020-09-25

**Authors:** Jessica G Wijesundara, Mayuko Ito Fukunaga, Jessica Ogarek, Bruce Barton, Lloyd Fisher, Peggy Preusse, Devi Sundaresan, Lawrence Garber, Kathleen M Mazor, Sarah L Cutrona

**Affiliations:** 1 Department of Population and Quantitative Health Sciences University of Massachusetts Medical School Worcester, MA United States; 2 Meyers Primary Care Institute Worcester, MA United States; 3 Department of Medicine University of Massachusetts Medical School Worcester, MA United States; 4 Center for Gerontology and Healthcare Research Brown University Providence, MA United States; 5 Department of Pediatrics University of Massachusetts Medical School Worcester, MA United States; 6 Reliant Medical Group Worcester, MA United States; 7 Health Services Research & Development, Center of Innovation Edith Nourse Rogers Memorial Hospital Veterans Health Administration Bedford, MA United States

**Keywords:** electronic health records, influenza vaccination, patient care, patient engagement

## Abstract

**Background:**

Patient reminders for influenza vaccination, delivered via an electronic health record patient portal and interactive voice response calls, offer an innovative approach to engaging patients and improving patient care.

**Objective:**

The goal of this study was to test the effectiveness of portal and interactive voice response outreach in improving rates of influenza vaccination by targeting patients in early September, shortly after vaccinations became available.

**Methods:**

Using electronic health record portal messages and interactive voice response calls promoting influenza vaccination, outreach was conducted in September 2015. Participants included adult patients within a large multispecialty group practice in central Massachusetts. Our main outcome was electronic health record–documented early influenza vaccination during the 2015-2016 influenza season, measured in November 2015. We randomly assigned all active portal users to 1 of 2 groups: (1) receiving a portal message promoting influenza vaccinations, listing upcoming clinics, and offering online scheduling of vaccination appointments (n=19,506) or (2) receiving usual care (n=19,505). We randomly assigned all portal nonusers to 1 of 2 groups: (1) receiving interactive voice response call (n=15,000) or (2) receiving usual care (n=43,596). The intervention also solicited patient self-reports on influenza vaccinations completed outside the clinic. Self-reported influenza vaccination data were uploaded into the electronic health records to increase the accuracy of existing provider-directed electronic health record clinical decision support (vaccination alerts) but were excluded from main analyses.

**Results:**

Among portal users, 28.4% (5549/19,506) of those randomized to receive messages and 27.1% (5294/19,505) of the usual care group had influenza vaccinations documented by November 2015 (*P*=.004). In multivariate analysis of portal users, message recipients were slightly more likely to have documented vaccinations when compared to the usual care group (OR 1.07, 95% CI 1.02-1.12). Among portal nonusers, 8.4% (1262/15,000) of those randomized to receive calls and 8.2% (3586/43,596) of usual care had documented vaccinations (*P*=.47), and multivariate analysis showed nonsignificant differences. Over half of portal messages sent were opened (10,112/19,479; 51.9%), and over half of interactive voice response calls placed (7599/14,984; 50.7%) reached their intended target, thus we attained similar levels of exposure to the messaging for both interventions. Among portal message recipients, 25.4% of message openers (2570/10,112) responded to a subsequent question on receipt of influenza vaccination; among interactive voice response recipients, 72.5% of those reached (5513/7599) responded to a similar question.

**Conclusions:**

Portal message outreach to a general primary care population achieved a small but statistically significant improvement in rates of influenza vaccination (OR 1.07, 95% CI 1.02-1.12). Interactive voice response calls did not significantly improve vaccination rates among portal nonusers (OR 1.03, 95% CI 0.96-1.10). Rates of patient engagement with both modalities were favorable.

**Trial Registration:**

ClinicalTrials.gov NCT02266277; https://clinicaltrials.gov/ct2/show/NCT02266277

## Introduction

Influenza infections contribute to increased health care costs and loss of productivity and can lead to serious complications and even death [1]. Effective strategies for the prevention of influenza are of critical importance as we enter the 2020-2021 influenza season. The confluence of the upcoming influenza season and the ongoing coronavirus disease 2019 (COVID-19) pandemic is expected to place additional stress on our health care system, fueled in part by similarities in presenting complaints for these two illnesses, as well as by the potential for increased risk of poor outcomes in patients co-infected with COVID-19 and influenza [2-4].

An estimated 5%-20% of the US population contracts influenza every year, with several hundred thousand people hospitalized annually due to influenza-related complications [5]. Estimates of annual influenza and pneumonia-associated deaths over the past decade reached as high as 61,000 in the 2017-2018 season [6-12]. According to Centers for Disease Control and Prevention (CDC) estimates, during the 2018-2019 influenza season, vaccinations prevented approximately 4.4 million flu illnesses, 58,000 hospitalizations, and 3500 deaths [13,14]. Since it takes approximately 2 weeks for antibodies to develop in response to influenza vaccination, the CDC recommends getting vaccinated before flu begins to spread within a community, by the end of October [15].

Despite widespread publicity promoting influenza vaccination, vaccines are underutilized [16-19]. In 2017, national vaccination coverage among adults for influenza was 37.1% [20], while the Healthy People 2020 target was 70% [21]. The CDC estimated flu vaccination coverage among adults aged ≥18 years as of mid-November 2018 was 44.9% [22]; at the end of the 2018-2019 influenza season, national influenza vaccination coverage was 45.3%[23].

Clinical decision support has been shown to improve health outcomes by supporting the delivery of timely, evidence-based and guideline-concordant medical care, including annual vaccinations [24-26]. Clinical decision support includes computerized reminders both to providers and patients. Many health systems effectively use provider-directed clinical decision support, including noninterruptive or interruptive (pop-up) alerts, reminding providers of recommended prevention or screening measures [27,28]. While frequently effective, provider-directed clinical decision support is subject to important limitations. If alerts are triggered by erroneous or incomplete electronic health record data, or if providers experience alert fatigue from an overwhelming number of notifications, alerts may be ignored or overridden [29-32]. Considering these challenges, and in the setting of nationwide adoption of electronic patient portals, patient-directed clinical decision support delivered via a patient portal offers an innovative approach to the promotion of timely influenza vaccination.

Electronic patient portals are secure websites that provide patients with 24-hour online access to limited electronic health record information. A portal provides patients with a personal health record that is tethered to their electronic health record. Accessible information within a tethered portal varies by health system but may include vaccinations, laboratory results, problem lists, allergies, and information from recent doctor visits or hospitalizations [33,34]. A core function of portals is secure messaging—electronic communication with the physician or health care team [35]. Patient portals have the potential to improve patient-provider communication, improve medication adherence, decrease office visits, increase self-management of disease and disease awareness, increase use of preventative medicine, and increase inclusion of patients in medical decision making [36-38]. Previous patient outreach interventions have been shown to improve rates of vaccination completion and have been tested using multiple options including mailed letters, postcards, live phone calls, automated phone messages, and combination postcard/phone-based [39,40]. Few studies have tested the use of patient-directed vaccination reminders sent via patient portals [25,26,41].

We conducted a randomized controlled trial aimed at improving rates of influenza vaccination among eligible adult patients in a large multispecialty group practice in central Massachusetts. We used electronic health record patient portal messages as well as interactive voice recognition calls to (1) promote early season influenza vaccination completion and (2) solicit patient self-report on vaccinations completed outside the clinic.

## Methods

### Study Objectives

The overarching goal of this study was to improve rates of early influenza vaccination (by the end of October) among eligible adults in an outpatient population. Our primary objective was to determine whether our outreach increased completion of influenza vaccinations and, if so, whether one mode of outreach was most effective. Additional objectives were to improve documentation of influenza vaccinations administered outside the practice by inviting patient self-report (improving the accuracy of existing decision support tools) and to track process measures (eg, rates of portal message opening and interactive voice response call answering).

### Study Design

We conducted a nonblinded randomized controlled intervention (NCT02266277) at a large multispecialty medical group in central Massachusetts. Previously, our team developed and tested interactive voice response and portal outreach which we targeted to patients who had no documented vaccination 2 months after the start of the season [25,26]; this study adapted our previous approach, targeting a broader population in early September in order to promote early vaccination and provide information on September and October flu clinic dates.

**Figure 1:**
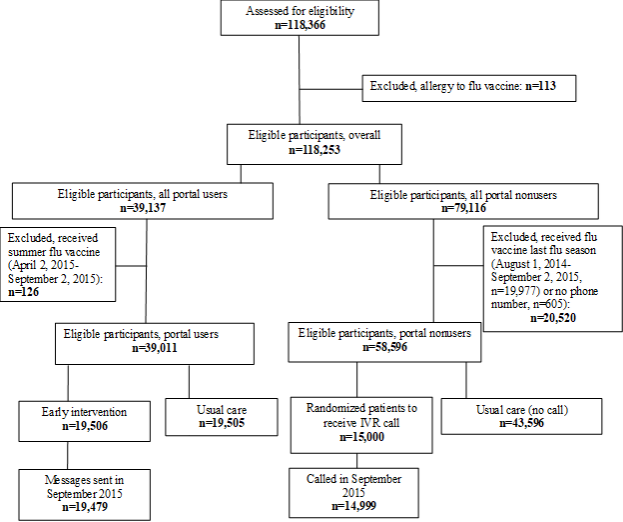
CONSORT (Consolidated Standards of Reporting Trials) Diagram describing randomized controlled trial to improve rates of early season influenza vaccinations using EHR portal messages and Interactive Voice Response calls.

Using a computer-generated randomization table, we assigned all active portal users to receive either a portal message promoting influenza vaccination, listing upcoming clinics, and offering online scheduling of vaccination appointments (n=19,506) or usual care with no portal message (n=19,505). Separately, we randomized all portal nonusers to receive either an interactive voice response call (n=15,000) or usual care with no interactive voice response call (n=43,596) (Figure 1). For portal users only, after the conclusion of the study and assessment of outcomes on November 3, messages were sent to the usual care group if they still did not have an electronic health record–documented influenza vaccination for the 2015-2016 season (n=14,118). The cost of calls prevented us from being able to send interactive voice response messages to the usual care group in the portal nonusers.

The study was reviewed and approved in 2014 by the Reliant Medical Group institutional review board; due to administrative changes, oversight was transferred to the University of Massachusetts institutional review board in 2015. A waiver for informed consent for patient outreach was approved by these institutional review boards. Patients were not compensated for their participation. The principal investigator (SC) oversaw the trial and data analysis.

### Study Population

#### Eligibility Criteria

Patients were eligible for the study if they (1) had a primary care provider at the medical group during the 12 months prior to randomization; (2) were aged ≥18 on the date of randomization; (3) had a recent office visit or telephone encounter with an internal medicine practitioner or family practitioner (defined as having had an office visit, phone encounter, consult, or complete physical exam within the 12 months prior to randomization). This requirement was intended to minimize inclusion of patients who had moved to another practice but whose names were retained in the medical group records. To ensure capture of patients transitioning from pediatric to adult care, the recent office visit could also be with a pediatrician.

A patient was eligible for inclusion in the electronic patient portal portion of the trial if the patient was an active user, which was defined as having an activated portal with a log-in at least once in the year preceding randomization.

#### Exclusion Criteria

For both interactive voice response and portal outreach, patients were excluded if there was electronic health record documentation of an allergy to influenza vaccines. For interactive voice response calls only, exclusion criteria also included the presence of any of the following on the date of randomization: (1) electronic health record documentation of influenza vaccination completion in the 2014-2015 influenza season (or documented influenza vaccination after the end of the 2014-2015 influenza season but before the start of the 2015-2016 season); (2) no listed phone number.

### Study Procedures

This study consisted of interactive voice response or portal-based outreach. Qualitative interviews conducted with patients, physicians, nurses, and staff informed the development of our outreach material [25,26].

### Electronic Patient Portal Intervention

We designed an outgoing secure portal message to be sent via patient portal to patients randomized to the portal message arm (Multimedia Appendix 1). Portal message content appeared in letter format with the signature line reflecting the name of the patient’s primary care provider. Portal messages were delivered through standard channels used for portal-based correspondence between the medical group’s health care providers and patients (ie, a generic message that contained neither personal health information nor any reference to vaccinations was delivered to patient’s email account; the message prompted patients to log in to secure portal account via hyperlink). Once logged in to the portal accounts, patients clicked on a message labeled “A Message from Your Primary Care Provider” to view the outreach message. The outreach message included information about upcoming flu clinics for September and October 2015.

Unique to the portal message (compared to interactive voice response phone messages) was the option of direct online scheduling of appointments for influenza vaccination. Information about accessing the CDC vaccination website appeared within the body of the portal message as a hyperlink (and was conveyed verbally in the interactive voice response script). Opportunities to report external influenza vaccinations and to report intent to get vaccinated matched the interactive voice response call content.

### Portal Message Delivery

Messages were sent out to 500 to 1500 patients daily over a period of 15 days in September 2015 in order to reduce the risk of being blocked by network bulk-spam filters.

### Interactive Voice Response Call Intervention

Interactive voice response calls appeared on caller ID as originating from the medical group. This is consistent with identification of interactive voice response calls used for appointment reminders at the time of the study. Combining voice response with branching logic, calls elicited patient self-reports of influenza vaccinations completed outside the medical group (Multimedia Appendix 2). For patients reporting no influenza vaccination completed, calls included information about upcoming flu clinics for September and October 2015. Patients reporting no influenza vaccination completed were also asked whether they intended to get vaccinated. Patients reporting that they were unsure or did not intend to get vaccinated were asked further questions on specific reasons why they did not plan to get a flu vaccination.

### Interactive Voice Response Call Delivery

Intervention interactive voice response calls, initiated on September 11, 2015, began by confirming that the person answering the phone was the intended patient recipient of the call. If voicemail was encountered or if the person reached was someone other than the patient, the interactive voice response system left a message asking patients to call back and provided an inbound call line number. The last interactive voice response outbound calls were placed on September 25, 2015. The inbound call line was maintained throughout the duration of outgoing calls and for 3 weeks after the final outgoing call was placed; patients who called this number from the phone number of record heard the interactive voice response call script in its entirety, beginning with questions confirming the identity of the caller.

### Study Outcomes

#### Primary Outcome

Our primary outcome was percentage of eligible patients with influenza vaccinations documented in the electronic health record as of November 3, 2015. We pulled data on vaccination rates as of this date, chosen in order to assess the impact of early outreach on completion of early immunization. Immunizations captured solely through the patient portal questionnaire or through the interactive voice response were excluded from the primary analysis in order to enable comparison with the control groups. The origins of all entered influenza vaccinations were tracked to allow our team to distinguish between sources of information on completed vaccinations.

#### Process Measures and Additional Outcomes of Interest

For portal messages, we calculated (1) percentage of recipients who logged in to the patient portal during early flu season (through November 3, 2015), (2) percentage of recipients who opened messages during early flu season, and (3) percentage of recipients who completed questionnaires. We tracked self-reports (via the portal) of influenza vaccinations completed. We also tracked patient-reported intent to get an influenza vaccination during the 2015-2016 flu season.

For interactive voice response calls, we calculated (1) percentage of recipients reached and (2) percentage of recipients who completed the calls by responding to questions. We tracked self-report (via interactive voice response) of influenza vaccinations completed. We also tracked patient-reported intent to get an influenza vaccination during the 2015-2016 flu season.

### Sample Size

With our proposed sample size, power calculations based on estimates of baseline vaccination rates indicated that 4286 participants per arm would give 80% power to detect a 3% improvement in influenza vaccination rates between groups (α=.05; 2-sided).

### Statistical Methods

#### Primary Outcome

To determine the impact of our interventions on early vaccination rates for the 2015-2016 influenza season, we calculated frequencies and performed intention-to-treat bivariate analyses of randomized patients, assessing whether vaccination completion was associated with group assignment. Due to different rates of electronic health record–recorded vaccination measured at baseline (in 2014) between portal users (35.9%) and nonusers (25%), and due to the differences in intervention (portal versus interactive voice response call), analyses for these groups were conducted separately.

We then performed multivariate logistic regression analyses, adjusting for demographic and practice-level covariates, we modeled the odds of receiving an influenza vaccination in the 2015-2016 influenza season.

## Results

### Baseline Characteristics

Baseline characteristics of both patient portal users and portal nonusers are reported in Table 1. Baseline characteristics were similar among portal users and portal nonusers. However, compared to the portal nonusers, portal users were more likely to be women, older, and have a higher level of health care utilization.

**Table 1:** Baseline characteristics of participants.

Characteristics		All (N=97,607), n (%)	Portal users		Portal nonusers	
			Portal message (n=19,506), n (%)	Usual care (n=19,505), n (%)	Call (n=15,000), n (%)	Usual care (n=43,596), n (%)
**Sex**						
	Female	53,250 (54.5)	12,230 (62.7)	12,249 (62.8)	7325 (48.8)	21,446 (49.2)
	Male	44,349 (45.4)	7275 (37.3)	7256 (37.2)	7672 (51.1)	22,146 (50.8)
	Missing	8 (0.01)	1 (0.0)	0 (0.0)	3 (0.0)	4 (0.0)
**Age**						
	18-34	30,556 (31.3)	4508 (23.1)	4597 (23.6)	5615 (37.4)	15,836 (36.3)
	35 - 49	25,579 (26.2)	5096 (26.1)	5062 (26.0)	3896 (26.0)	11,525 (26.4)
	50 - 64	26,495 (27.1)	6275 (32.2)	6224 (31.9)	3547 (23.6)	10,449 (24.0)
	65-74	8593 (8.8)	2419 (12.4)	2388 (12.2)	965 (6.4)	2821 (6.5)
	75+	6384 (6.5)	1208 (6.2)	1234 (6.3)	977 (6.5)	2965 (6.8)
**Race**						
	White	66,020 (67.6)	14,180 (72.7)	14,223 (72.9)	9608 (64.1)	28,009 (64.2)
	Black	3417 (3.5)	456 (2.3)	440 (2.3)	630 (4.2)	1891 (4.3)
	Asian	3502 (3.6)	737 (3.8)	715 (3.7)	528 (3.5)	1522 (3.5)
	American Indian or Alaska Native	1021 (1.1)	140 (0.7)	144 (0.7)	194 (1.3)	543 (1.2)
	Other	10,366 (10.6)	1672 (8.6)	1745 (8.9)	1769 (11.8)	5180 (11.9)
	Missing	13,281 (13.6)	2321 (11.9)	2238 (11.5)	2271 (15.1)	6451 (14.8)
**Health care utilization level**						
	Had office visit^a^	80,748 (82.7)	17,896 (91.7)	17,908 (91.8)	11,609 (77.4)	33,335 (76.5)
	Did not have office visit	16,859 (17.3)	1610 (8.3)	1597 (8.2)	3391 (22.6)	10,261 (23.5)

^a^12 months prior to randomization.

### Portal Users

Among portal users, 28.4% (5549/19,506) of message recipients and 27.1% (5294/19,505) of the usual care group had documentation in their electronic health records that they received that influenza vaccinations on or before November 3, 2015 (*P*=.004). Portal users who received the messages were significantly more likely to have received the influenza vaccination compared to the usual care group (odds ratio [OR] 1.07, 95% CI 1.02-1.12). This finding was consistent even after adjusting for age, race, sex, and health care utilization (OR 1.07, 95% CI 1.02-1.12) (Table 2).

**Table 2:** Likelihood of receiving an early-season influenza vaccination.

Recipients	n	Unadjusted		Adjusted^a^	
		OR^b^	95% CI	OR	95% CI
Portal message^c^	39,011	1.07	(1.02, 1.12)	1.07	(1.02, 1.12)
Interactive voice response call^c^	58,596	1.03	(0.96, 1.10)	1.03	(0.96, 1.11)

^a^Adjusted for age, sex, race, and health care utilization level (where utilization was defined as office visit, phone encounter, consult, or complete physical exam within the 12 months prior to randomization).

^b^OR: odds ratio.

^c^The reference is usual care.

### Portal Nonusers

Among portal nonusers, 8.4% (1262/15,000) of call recipients and 8.2% (3586/43,596) of usual care recipients received vaccinations (*P*=.47). Bivariate and multivariate analysis showed nonsignificant differences in influenza vaccination rates between intervention and usual care groups (Table 2).

### Process Measures

#### Portal Message

Among patient portal message recipients, 71.2% (13,862 recipients out of 19,479 to whom the message was sent) logged in to the patient portal on or after the date of message delivery through the end of early flu season (September 9, 2015 to November 3, 2015). Messages were opened by 51.9% (10,112/19,479) recipients; 13.2% (2570/19,479) responded to the first question asking if they received a flu vaccination on or after August 1, 2015, 2.0% (386/19,479) reported already receiving a vaccination and 11.2% (2176/19,479) responded to a second question assessing their intention to receive a flu vaccination during the flu season (asked only of those who were not already vaccinated) (Table 3).

Of those opening messages, 25.4% (2570/10,112) responded to our question on receipt of influenza vaccination, 3.8% (386/10,112) reported already receiving vaccinations and 21.5% (2176/10,112) responded to our question on whether they planned to get vaccinated.

**Table 3:** Process measures and self-reported influenza vaccinations for portal message recipients.

Action	Portal users, n (%)
Randomized	19,506
Message sent^a^	19,479 (100)
Logged in to patient portal	13,862 (71.2)
Opened message	10,112 (51.9)
Responded to “Have you received a flu vaccination on or after August 1, 2015?”	2570 (13.2)
Reported receiving a flu vaccination	386 (2.0)
Responded to “Do you plan to get a flu vaccination this flu season?”	2176 (11.2)
Reported that they planned to get a flu vaccination	1814 (9.3)

^a^27 patients were no longer portal users by the time we sent the messages (due to change in medical record numbers, invalid patient portal IDs, etc).

### Interactive Voice Response Call

Among interactive voice response call recipients, 50.7% of patients were reached (7599/14,984); 36.8% (5513/14,984) responded to the first question asking if they received a flu vaccination on or after August 1, 2015, 3.8% (575/14,984) reported receiving their vaccination and 24.1% (3613/14,984) responded to a second question assessing their intention to receive a flu vaccination during the flu season (asked only of those who were not already vaccinated) (Table 4).

**Table 4:** Process measures and self-reported influenza vaccinations for interactive voice response call recipients.

Action	Call recipients, n (%)
Randomized	14,999
Call attempted^a^	14,984 (100)
Target reached (inbound and outbound calls)	7599 (50.7)
Responded to “Have you received a flu vaccination on or after August 1, 2015?”	5513 (36.8)
Reported receiving a flu vaccination	575 (3.8)
Responded to “Do you plan to get a flu vaccination this flu season?”	3613 (24.1)
Reported that they planned to get a flu vaccination	1415 (9.4)

^a^15 patients had invalid/blank phone numbers, or the patient was on a “Do Not Call” list, by the time we placed the calls.

Of those reached, 72.5% (5513/7599) responded to our question on receipt of influenza vaccination, 7.6% (575/7599) reported already receiving a vaccination, and 47.5% (3613/7599) responded to our question on whether they planned to get vaccinated.

## Discussion

### Principal Results

Our study showed a clinically small but statistically significant improvement in completion of early season influenza vaccinations among those randomized to receive outreach via patient portal, compared to a usual care control group (OR 1.07, 95% CI 1.02-1.12). There was no significant increase of early season influenza vaccinations among those randomized to receive an interactive voice response call (OR 1.03, 95% CI 0.96-1.10).

This outreach was designed to deliver a relatively time-sensitive message (eg, reminding patients of the importance of influenza vaccination and alerting them of upcoming vaccination clinics in September and October), and as such, represents a successful, brief patient engagement effort. We attained greater than 50% opening rates for portal messages and reached the targeted patient on over 50% of interactive voice response calls, achieving similar levels of exposure to the messaging for the two interventions. Among portal message recipients, more than one quarter of those who opened the message responded to our subsequent question on whether they had received or intended to receive their influenza vaccination. Among interactive voice response call recipients, close to three-quarters of those reached responded to the question.

A small number of patients reported influenza vaccinations that had been completed in the community; data on patient-reported vaccinations performed in the community were then uploaded into the electronic health record in order to improve the accuracy of existing influenza vaccination alerts directed at primary care providers. The relatively small number of self-reported flu vaccinations (961/20,978 participants across the portal message intervention and the interactive voice response intervention arms combined) has several possible explanations. It is possible that participants who had already been vaccinated found the outreach less relevant; and therefore, chose not to engage (eg, chose not to open the message or chose to hang up the phone). Relatively low rates of self-reported vaccination completion may also be attributable to the timing of our outreach—intentionally positioned at the start of the flu season. In contrast, our past work [26] captured much higher proportions of self-reported vaccinations (2591/20,000 intervention patients) when participants were approached several months past the start of flu season, which highlights the importance of asking patients to self-report vaccinations administered outside the clinic.

Although these “discovered” immunizations were incorporated into the electronic health record, they were not counted in the primary analysis; this allowed comparison with the usual care groups who were not queried for outside immunizations. While recognizing that the actual immunization rate was higher than the documented rate, any differences in the documented rate between intervention and control groups can reasonably be attributed to increased vaccination rates. Data on intention to be vaccinated were also elicited. Studies have shown that stating intent to complete a behavior can enhance likelihood of follow-through [42,43], thus it is possible that engaging patients in this manner may have contributed to the modest success of our intervention.

Once designed, portal messages (sent out in several batches) required minimal staff time to deliver. Our messages were designed to be easily adapted for use in future years; our messages have already been adapted and implemented by our medical center to target high-risk populations (eg, children with asthma).

### Comparison With Prior Work

There have been multiple studies [44-47] documenting success in use of patient-directed reminders for influenza vaccination. Effective reminders described include letters, telephone invitations, phone calls from a peer, tailored communications, customized letters/phone calls, and client-based appraisals [44]. Text message reminders have been shown to be successful for influenza vaccination reminders in pediatric and adolescent populations [45], in patients with rare diseases [46], and among high-risk patients [47].

While portal-based outreach directed at patients has been described for a variety of preventive measures [48], few studies have tested use of patient-directed influenza vaccination reminders sent via patient portals; one study [49] that has done so focused exclusively on untethered (ie, personally controlled) patient health records among a university student and staff population in Australia, yielding a 6.7% greater likelihood of influenza vaccination among users of the personal health records compared to those randomized to a 6-month waitlist for portal activation [49]. In our past work [25,26], we tested similar portal and interactive voice response outreach messages using a factorial design and targeting a population more likely to be nonadherent to vaccination guidelines (patients who had no documented vaccination 2 months after the start of the flu season). In that study [25,26], we found small but statistically significant improvements in influenza vaccination rates among recipients of either outreach method compared to usual care (portal message alone (OR 1.20, 95% CI 1.06-1.35); interactive voice response call alone (OR 1.15, 95 % CI 1.02-1.30); both messages (OR 1.29, 97.5% CI 1.13-1.48). In their recent randomized trial, Szilagyi et al [41] studied the effect of 1, 2, or 3 patient portal reminders on influenza vaccination rates among 164,205 patients in 52 primary care practices. They noted small but statistically significant improvements in vaccination rates with an attenuating effect of repeated messaging (37.5% for those receiving no reminders; 38.0% for 1 reminder; 38.2% for 2 reminders, and 38.2% for 3 reminders). Higher overall rates of vaccination were documented in their trial in comparison to ours; this is likely due in part to the study’s inclusion of children (who have a higher rate of influenza vaccination than that of the overall adult population) as well as their measurement of vaccination rates at the end of the influenza season (March 31).

The rates of patient engagement (measured by message opening and subsequent action) demonstrated by our patient portal recipients were comparable to those from previously published patient portal outreach studies. In our previous influenza outreach intervention [25,26] (in a population unvaccinated 2 months into the flu season), messages were opened by slightly more than half of message recipients and interactive voice response targets were reached in just over 60%. In that prior study, among those who opened portal messages, 28.6% responded to subsequent questions. Among those reached via interactive voice response call, 78.3% responded to questions. In their patient portal intervention aimed at improving influenza vaccination rates, Szilagyi and colleagues [41] found opening rates of 52.9% among patients receiving a single portal message (increasing to 55.9% in a 2-reminder group and 58.8% in a 3-reminder group).

Fischer and colleagues [50] found that among patients randomized to active reminders about multiple health maintenance services (eg, hemoglobin A_1c_ testing, lipids, etc), nearly 65% of patients logged in to the portal after receiving the first of several messages. In a study [51] of colorectal cancer screening reminders delivered via patient portal, among the 552 patients randomized to receive messages, 54% viewed the message and 9% performed a suggested web-based risk assessment tool. In a study describing the reach and feasibility of an interactive lung cancer screening decision aid delivered by patient portal, Dharod and colleagues [52] found that 86% of lung cancer screening eligible patients identified by an electronic health record algorithm to receive a patient portal message read the message, 40% then visited a web-based decision aid for lung cancer screening, and 35% completed questionnaires to determine their eligibility for lung cancer screening.

### Limitations

Our study had several limitations. Despite responding to inquiries on flu vaccinations receipt at higher rates, the interactive voice response group did not show a significant impact on vaccination rates (while the portal message group showed a small statistically significant improvement in vaccination rates). This discrepancy could be due in part to differences in the baseline characteristics of portal nonusers compared to portal users. For several reasons, interactive voice response call recipients may have been more resistant to completion of influenza vaccination than portal message recipients. Portal nonusers were less likely to have had an office visit in the previous 12 months and might have been less actively engaged in their health care overall. In addition, eligibility for the interactive voice response study was intentionally more stringent, with patients included only if they lacked documentation of an influenza vaccination in the previous flu season. This study design choice was made with guidance from the medical center where our study was implemented and was intended to make available the limited resource of interactive voice response calls to the broadest possible number of nonadherent patients (our study covered the cost for only 15,000 calls but we had no limit on the number of portal messages that we could send). It is possible that the interactive voice response calls might have yielded a greater impact if we had not opted to employ this more stringent eligibility criterion.

### Conclusions

There are compelling reasons to use existing functionality within electronic health record–tethered portals to promote influenza vaccination. For vaccination outreach, data recorded in the electronic health record through routine care delivery can inform real-time identification of unvaccinated populations. Portal-based outreach can be more cost-effective than phone calls or mailings and easier to implement than a new app; simple messages can be sent out by office staff without additional informatics expertise. Studies [37,38,53,54] show that patient portals can enhance patient empowerment and sense of autonomy, enhance patient engagement, improve medication adherence, decrease office visits, increase self-management of disease and disease awareness, increase use of preventive medicine, and increase inclusion of patients in medical decision making.

Our parallel interventions to patient portal users and portal nonusers allowed us to assess the impact of outreach supporting influenza vaccination in both groups. Our study demonstrated a small improvement in influenza vaccination rates among portal message recipients, and successful patient engagement in both portal message recipients and interactive voice response call recipients. This method is readily applicable to current practice.

As we simultaneously face the upcoming influenza season and the ongoing COVID-19 pandemic, expanding influenza vaccination coverage in ambulatory populations can decrease the strain on our overtaxed health system and may help avert poor outcomes in patients at risk for co-infection with influenza and COVID-19.

Our findings of a small but significant improvement in influenza vaccination rates resulting from portal-based outreach represent an important contribution to the national conversation on caring for and protecting our patients in the upcoming months and for years to come.

## Appendix

Multimedia Appendix 1 Example of portal outreach message.

Multimedia Appendix 2 Example of interactive voice response call script.

## Abbreviations

CDC: Centers for Disease Control and Prevention
OR: odds ratio
