# Supplementary material for: Electronic Health Record Portal Messages and Interactive Voice Response Calls to Improve Rates of Early Season Influenza Vaccination: Randomized Controlled Trial
Source: J Med Internet Res. 2020 Sep 25;22(9):e16373. doi: 10.2196/16373 (PMC7547389; doi:10.2196/16373)
Supplement: Multimedia Appendix 1 [file jmir_v22i9e16373_app1.pdf]

Appendix 1. Example of Portal Outreach Message

Subject: A Message from Your Primary Care Provider

From: [Patient's Specific PCP]

Tasks: Brief Flu Vaccine Questionnaire [clickable link]

Flu season is coming soon. Even healthy people can get sick enough to miss school or work and can spread the flu to family and friends. Sometimes people with the flu get very sick and need to be hospitalized.

To help protect you and your family, we recommend the flu vaccine for everyone aged 6 months and older. Please complete the attached questionnaire (just 2 questions) to tell us if you've already been vaccinated and when. Simply click on the "Brief Flu Vaccine Questionnaire" link above.

If you still need a flu vaccine, you can do any of the following:

- Stop by Reliant Medical's flu clinics (scroll down for locations, dates, and times).
- Schedule an appointment for a flu vaccine by calling our office at [PCP's specific number].
- Use the patient portal to request an appointment. If you want, you can use your mobile device to do this. After you request an appointment, we will call you back to confirm the date and time.
- Ask for your flu vaccine at your next doctor's appointment.

To learn more about the flu vaccine, click here:
http://www.cdc.gov/flu/pdf/freeresources/general/no-excuses-flu-vaccine.pdf AND
http://www.cdc.gov/flu/

[If patient has pneumo flag] At your next appointment, please also ask us if you need a pneumococcal vaccine, sometimes called a pneumonia vaccine. This vaccine helps protect against infections that can lead to pneumonia and other serious infections and illnesses.

To learn more about the pneumococcal vaccine, click here: http://www.cdc.gov/pneumococcal/

Scroll to the top to complete the questionnaire attached as a task.

Adult Flu Clinics for September/October 2015

[Specific Clinic Location]: Saturday, September 26th from 7:30am-11:30am
[Specific Clinic Location]: Saturday, September 26th from 8:00am-12:00pm
[Specific Clinic Location]: Wednesday, October 7th from 5:00pm-7:00pm
[Specific Clinic Location]: Saturday, October 10th from 9:00am-12:00pm
[Specific Clinic Location]: Saturday, October 10th from 8:00am-12:00pm
[Specific Clinic Location]: Saturday, October 17th from 9:00am-1:00pm

[Specific Clinic Location]: Saturday, October 17th from 9:00am-12:00pm
[Specific Clinic Location]: Saturday, October 17th from 9:00am-12:00pm
[Specific Clinic Location]: Saturday, October 17th from 9:00am-1:00pm
[Specific Clinic Location]: Saturday, October 24th from 9:00am-1:00pm
[Specific Clinic Location]: Saturday, October 24th from 9:00am-12:00pm
[Specific Clinic Location]: Tuesday evenings (9/22; 9/29; 10/6; 10/13; 10/20; 10/27 – please book appointment) from 5-7 pm

Sincerely,

[Specific PCP's name]

"Brief Questionnaire" in title

Have you received a flu vaccine on or after August 1, 2015?

☐ Yes

☐ No

[If yes to 'Have you received a flu vaccine,' display:]

So we may update your record, please enter the month, if you know it, on which you were vaccinated:
[Please select month:]
August 2015
September 2015
October 2015
November 2015
December 2015
I do not know

So we may update your record, please enter the day, if you know it, on which you were vaccinated:
Day_________(dropdown)

[If no to 'Have you received a flu vaccine,' display:]

Do you plan to get a flu vaccine this flu season?

☐ Yes

☐ No

[If yes to Do you plan to get a flu vaccine this flu season? *display:]*

That's good to know. Getting a flu vaccine only takes a few minutes. Reliant offers many convenient options, which are included in your MyChart message. Please let your doctor's office know when you've gotten your vaccine so that we may update your records.

Some flu facts:

The flu vaccine is safe.
Flu vaccines have been given to millions of people for more than 50 years and have a very good safety record. Each year, Centers for Disease Control (CDC) works closely with the U.S. Food and Drug Administration (FDA), and other partners to ensure the highest safety standards for flu vaccines.

The most common side effects of flu vaccines are mild.
The flu vaccine cannot cause flu illness; however, it can cause mild side effects that may be mistaken for flu. For example, people vaccinated with the flu shot may feel achy and may have a sore arm where the shot was given. People vaccinated with the nasal spray flu vaccine may have a stuffy nose and sore throat. These side effects are NOT the flu. If experienced at all, these effects are usually mild and last only 1-2 days.

Some people feel ill after receiving the flu vaccine because they were exposed to flu viruses shortly before getting vaccinated or during the two weeks after vaccination (while immune protection develops). We encourage everyone to get vaccinated early, before flu begins to spread.

What happens to this information? We periodically evaluate our programs for effectiveness and are trying to learn how to better serve our patient's needs for preventive care. This questionnaire is voluntary and your responses will be entered into your Reliant medical record. If you have received the flu vaccine this year, responding to the questionnaire will help keep your records up to date.

[If no to Do you plan to get a flu vaccine this flu season? *display:]*

Thank you for letting us know you don't plan to get vaccinated this flu season. People your age do get vaccinated against the flu each year. If you would like to learn more about the flu vaccine before making your final decision, below are some flu facts that might be helpful.

The flu vaccine is safe.
Flu vaccines have been given to millions of people for more than 50 years and have a very good safety record. Each year, Centers for Disease Control (CDC) works closely with the U.S. Food and Drug Administration (FDA), and other partners to ensure the highest safety standards for flu vaccines.

The most common side effects of flu vaccines are mild.
The flu vaccine cannot cause flu illness; however, it can cause mild side effects that may be mistaken for flu. For example, people vaccinated with the flu shot may feel achy and may have a sore arm where the shot was given. People vaccinated with the nasal spray flu vaccine may have a stuffy nose and sore throat. These side effects are NOT the flu. If experienced at all, these effects are usually mild and last only 1-2 days.

Some people feel ill after receiving the flu vaccine because they were exposed to flu viruses shortly before getting vaccinated or during the two weeks after vaccination (while immune protection develops). We encourage everyone to get vaccinated early, before flu begins to spread.

Getting a flu vaccine only takes a few minutes. Reliant offers many convenient options, which are included in your MyChart message. Please let your doctor's office know if you get your vaccine so that we may update your records.

What happens to this information? We periodically evaluate our programs for effectiveness and are trying to learn how to better serve our patient's needs for preventive care. This questionnaire is voluntary and your responses will be entered into your Reliant medical record. If you have received the flu vaccine this year, responding to the questionnaire will help keep your records up to date.
