# Supplementary material for: Electronic Health Record Portal Messages and Interactive Voice Response Calls to Improve Rates of Early Season Influenza Vaccination: Randomized Controlled Trial
Source: J Med Internet Res. 2020 Sep 25;22(9):e16373. doi: 10.2196/16373 (PMC7547389; doi:10.2196/16373)
Supplement: Multimedia Appendix 2 [file jmir_v22i9e16373_app2.pdf]

Appendix 2. Example Submodules of Interactive Voice Recognition (IVR) script for outgoing call (adapted from IVR script):

Example 1: Patient receiving outgoing call is believed to be overdue for pneumococcal vaccine in addition to influenza vaccine, based on Medical Group EHR data. Upon receiving the call, the patient reports he/she has gotten a flu vaccine outside of the Medical Group and is able to report month, date, and location. Information is provided on pneumococcal vaccination as well.

Hello, this is Reliant Medical Group, calling for [patient]. Yes or no, is this [he/she]?

[If yes:] We're helping to make sure that our patients get their flu vaccines. Have you had a flu vaccine any time after August 1st of this year?

> [If yes:] Terrific! And yes or no, do you remember the month you had the flu shot?
>
> [If yes:] So that we can update our records, please tell us the month, now.
>
> [month given]
>
> Thanks. It'd be great to know the exact date too – for example, the 23rd. So if you can remember please tell me the date you received your flu vaccine, now, or you can say don't remember.
>
> [date given]
>
>> Thank you. We're glad you're taking steps to protect your health. Share your decision with a friend or family member – remind them to get their flu vaccine too!
>
> Also, please ask your doctor if you need a pneumococcal vaccine, sometimes called a pneumonia vaccine. This vaccine helps protect against infections that can lead to pneumonia and other serious infections and illnesses. Do you plan on speaking with your doctor about having this vaccine?
>
> [If yes:] That's great! In the meantime, if you want to learn more, you can go online to www.cdc.gov/pneumococcal. Pneumococcal is spelled P-n-e-u-m-o-c-o-c-c-a-l.
>
> Your Reliant doctors, nurses, and office staff believe the flu and pneumonia vaccines are important. And many people your age also protect themselves and the people they care about by getting vaccinated. To learn more about the flu vaccine, just go to www.cdc.gov. Thank you for your time. Have a good evening. Goodbye.

Example 2: Outgoing call was placed and message was left on answering machine. Patient calls back and encounters the message for an incoming call. Based on Medical Group EHR data, this patient is believed to be overdue for influenza vaccine, but is already up to date with pneumococcal vaccination. Upon receiving the call, the patient confirms that he/she has not received an influenza vaccine this year. This patient will be offered information on upcoming clinics and will initially decline. Questions on barriers to influenza vaccination will be asked, following which the patient will be asked about intent to get the influenza vaccination. In this example, the patient now answers yes, he or she intends to get vaccinated, and will be provided with information on options for receiving this vaccine.

Hello! Thank you for calling in to receive an important health reminder from Reliant Medical Group.

We're helping to make sure that our patients get their flu vaccines. Have you had a flu vaccine any time after August 1st of this year?

[If no]

Okay. If you'd like we can tell you several easy ways to get your vaccine through Reliant Medical Group. Would you like to hear about these now?

[If no]

All right. Are you planning to get a flu vaccine?

[If no]

Even healthy people can get sick enough to miss school or work and can spread the flu to family and friends. Sometimes people with the flu get very sick and need to be hospitalized. To help protect you and your family, we recommend the flu vaccine for everyone aged 6 months and older. We'd like to ask just a few questions to help us understand if there is a specific reason why you don't plan to get a flu vaccine. [*If patient answers no for these barrier questions, automated caller answers 'OK' or 'All right' and moves to next barrier question]*

Some of our patients believe the flu vaccine causes side effects. Is this true for you?

[If yes] That's good to know. The flu vaccine can cause mild side effects that may be mistaken for the flu. For example, people vaccinated with the flu shot may feel achy or have a sore arm where the shot was given. People vaccinated with the nasal spray flu vaccine may have a stuffy nose and sore throat. It's important to know that these side effects are not the flu and if felt at all, are usually mild and last 1 to 2 days.

Do you believe that the flu vaccine causes the flu?

[If yes]. All right. A lot of people feel this way. But the truth is the flu vaccine cannot give you the flu. Some people do get the flu after receiving the flu vaccine, but that's

because they were exposed to the flu before they got their vaccine, or during the two weeks it can take for the vaccine to take effect. So we encourage you to get vaccinated early, before the flu begins to spread.

Do you have other worries about the safety of the vaccine?

[If yes] Okay. You may be interested to hear that flu vaccines have been given for over 50 years to millions of people and have a very good safety record. The Centers for Disease Control, or the CDC, works closely with the Food and Drug Administration, the FDA, to make sure flu vaccines meet high safety standards. We encourage you to follow-up with your doctor because they can answer any specific questions you have about the flu vaccine.

Now that you may know a little more about why having a flu shot is such an important part of protecting yourself from getting sick, we'd like to ask again – will you get a flu shot this year?

[If yes] Would you like to hear about the different options you have for getting you flu vaccine through Reliant Medical Group?

[If yes] All right. Would you like me to wait a moment while you get a pen and some paper?

[If yes] Okay. I'll hold on the line, and when you're ready, please say 'hello'. Are you ready to get started?

[If yes] Reliant offers flu clinics most Saturday mornings from September 26th through October 24th and also offers some evening clinics on weekdays. Visit our website at [website] for locations and further details. The [name] flu clinic is on [date, from starting time to ending time]. Or you can schedule an appointment for a flu vaccine either by calling your Reliant doctor's office. You can also ask for a flu vaccine at your next regularly scheduled appointment.

Would you like me to repeat that?

[If no] Your Reliant doctors, nurses, and office staff believe the flu vaccine is important and they get vaccinated every year. Many people your age also protect themselves and the people they care about by getting vaccinated. To learn more about the flu vaccine, just go to www.cdc.gov/flu. Thank you for your time. Have a good day. Goodbye.
